# The conundrum of delivering nutrition benefits, mitigating risks, and avoiding inertia

**DOI:** 10.1093/ajcn/nqab272

**Published:** 2021-09-02

**Authors:** Lynnette M Neufeld, Mduduzi N N Mbuya

**Affiliations:** Global Alliance for Improved Nutrition, Geneva, Switzerland; Global Alliance for Improved Nutrition, Washington, DC, USA

See corresponding perspectives on pages 1257 and 1261.

We read with interest 2 perspective pieces (1, 2) that appeared in previous issues. Both articles provide combinations of evidence reviews and opinions of the authors that are interesting and provocative in their interpretation of the evidence and in their reflections on its implications for policy and programs. We are pleased to see the *American Journal of Clinical Nutrition* create the space for such reflection, thus providing an important channel for the nutrition community to grapple with the complexity of our field and the apparent contradictions in evidence, and we appreciate the opportunity to add our reflections to the mix.

One of the perspectives calls for stronger and explicit commitments to fortified products (or supplementation) for a specific target population (infants and young children) (1), justified primarily by the lack of progress in addressing anemia and iron deficiency in this group. The other calls for much more restraint in the implementation of actions, particularly fortification, in response to the lack of progress in addressing anemia and iron deficiency in groups including but not limited to infants and young children (2). An outsider to the nutrition community might consider this a rather perplexing conundrum—do we need more programmatic action, or do we need more restraint? Although they will not resolve this apparent contradiction, we reflect here on 4 questions—the responses to which may be helpful to increase understanding of how and why it exists, and what we as the nutrition community might do to help navigate such apparent contradictions.

The first question is what considerations should be brought to bear in evaluating, interpreting, and using nutrition evidence to make contextually relevant programmatic and policy recommendations with impact potential?

Both papers provide reflections based on the biology of nutrition (e.g., better/more nuanced understanding of nutritional needs and consequences of nutritional deficiencies). However, the arguments of the authors in both articles extend to economics, ethics, and policy and planning. The authors call for actions embedded in policy making, advocacy, financial allocations, and beyond. These reflections are reminiscent of a series of discussions from the 1990s. In a letter to the editor published in the *Journal of Nutrition*, Mason and colleagues (3) address the low and tenuous nature of nutrition funding and the prioritization of limited resources by calling for a transdisciplinary view of “public nutrition.” An earlier article (4) distinguished perceived funding and prioritization problems from a need for nutrition engineers—who combine an understanding of the etiology and consequences of malnutrition with training in program operations. These and the various articles in response (5, 6) are worth (re-)reading, mainly because of their emphasis on training, illustrated in Tagwireyi's (7) observation that “universities should continuously ask themselves whether their programs are equipping people to provide the service required of them..” This point was further emphasized by Jean Pierre Habicht in 1999 (8):

“My hope is that 15 years from now every public nutritionist will also have exposure to enough economics to understand its use as a descriptor and predictor of behaviour and as a tool for improving nutrition, that there will be a discipline of nutrition economics (not just food economics), and that nutrition policy will become a scientific discipline.” And further “…development of a feasible curriculum at different levels of training that incorporates the necessary knowledge across the breadth of disciplines to produce both the nutrition engineers and the nutritional sciences necessary to train and support the engineers.” (JP Habicht)

We are now 22 years on from the publication of these observations, and while we believe there has been progress, we cannot claim success based on Habicht's hope! These gaps are still constraining our ability to influence policy and programmatic processes and ensure that the evidence we generate, consolidate, and publish is comprehensive and translated to actionable recommendations that resonate with policy makers.

The second question is, how do we make sense of the complexity of our discipline, so we can better engage with other disciplines toward coherent and comprehensive policy and program recommendations?

The UNICEF conceptual framework of the determinants of malnutrition (9) is well known (**Figure 1**). This figure, for the first time, articulated the complexity and multidimensionality of nutrition in a clear, comprehensible, logical, and evidence-informed manner. It sharpened our focus on where, why, and how to act to improve nutrition, and it continues to influence research priorities and design, program design and evaluation, and sometimes, even donor funding priorities. But the level of familiarity that has ensued from this figure may jade us to the extremely complex implications of what it actually means to accomplish the lofty goals to reduce “all forms of malnutrition.” Nothing less than the eradication of poverty, and alignment of political, economic, and social goals, will do. This brings us back to the first point: the diversity of skills and action needed to address all forms of malnutrition go far beyond our nutrition training, and we have much to do still to obtain the political will, economic commitments, and all the rest that is needed to address the determinants of malnutrition in ways that both treat and support those with immediate need, but also prevent and reverse current trends. Which brings us to the third question…

**FIGURE 1:**
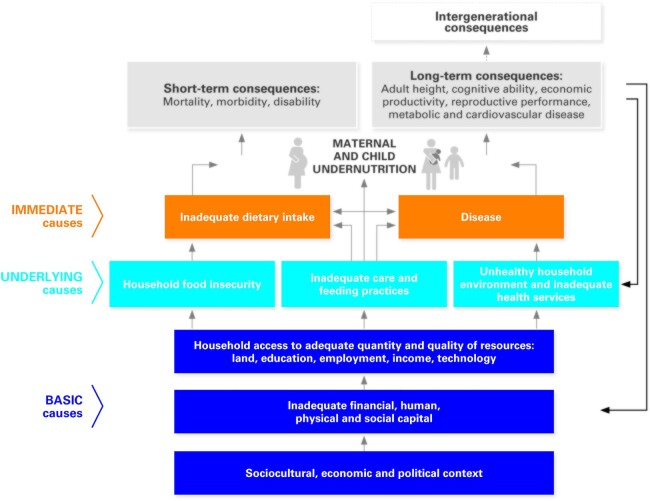
UNICEF conceptual framework of the determinants of malnutrition. Reproduced with permission from (9).

As a single sector, we cannot alone “catch the rainbows” as might be needed to once and for all, eradicate all forms of malnutrition. So how do we find and keep the focus where there is most potential for impact?

In nutrition much or most of the evidence [e.g., (10)] and global guidance (such as the World Health Organization nutrition guidelines) focuses our efforts on the immediate causes and consequences of malnutrition (the upper portion of the UNICEF conceptual framework). This evidence guides us to specific interventions to improve dietary intake, often of a single or several nutrients lacking in the diet, or single nutrition issues (e.g., anemia). Some say that this makes nutrition a highly reductionist field. But our experience suggests that this is exactly what many governments and donors want to buy with their funds earmarked for nutrition. Who can blame them? Evidence is robust, results are tangible, and we have convinced them—rightly—that lives can be saved in this manner. Zlotkin and Dewey make a strong plea urging that this focus not be lost. At the same time, Kurpad et al. suggest that we have not always been as coordinated as we should be, even within efforts to fix these specific nutrition issues, and that in doing so, at least in the case they propose from India, potential risks, and many sociocultural, economic, ethical and political considerations (as illustrated in the lower half of the UNICEF framework) are insufficiently brought to bear.

Given these apparent contradictions, the fourth question we would like to raise is how can both perspectives be right, and how can we enhance the ability of program planners to make informed decisions?

We as the nutrition community can do much to resolve this conundrum. First, there are fundamental biological questions that still constrain the quality of recommendations to address several nutrition issues—anemia is certainly one of those, since nonnutritional causes are important of course. We must resolve these evidence gaps and accelerate the translation of the complex evidence [e.g., (11)] that is likely to emerge. Second, the further down the conceptual framework we go, the weaker grows the evidence base on how actions lead to meaningful nutrition improvements, making the case for investment challenging, and the risk for inconsistent and muddled messages of policy and program priorities very real. We need to fix that by generating the evidence and recognizing that innovation in methodologies is required to do this well. Finally, in supporting policy and programmatic processes, we must never lose sight of the principles of good design, implementation, and measurement laid out through the UNICEF framework, WHO guidance [e.g., (12)], and tools and good practice resources. These resources provide the basis for programs that are driven by demonstrated need, through elucidation of biologically plausible pathways to impact, an understanding of implementation feasibility, and must be continually reviewed to ensure that benefits are delivered (the ethical questions of beneficence and justice in beneficence raised by Zlotkin & Dewey), and risks avoided (the ethical question of malfeasance raised by Kurpad et al.). If we get these points right, then the right linkages between biology, policy and economics can be made, and course corrective actions relied upon so that fears of plausible future risks do not result in inertia today. At the end of the day, this is our ethical imperative.

## Notes

The authors reported no funding received for this study.
